# Topics of Public Interest

**Published:** 1909-02

**Authors:** 


### TOPICS OF PUBLIC INTEREST.

### Committee to Fight Tuberculosis in Buffalo is Permanently Organised.

**Thomas B. Lockwood its President—Must have a Hospital—Mayor Adam, it is said, will soon give Hearing on Proposition to Bond the City.**

[*Buffalo Express*, January 5, 1909.]

A REPRESENTATIVE body of men and women which will combat tuberculosis in this city met at the Hotel Iroquois yesterday afternoon and completed a permanent organisation. It will be known as the Buffalo Association for the Relief and Control of Tuberculosis. The meeting was brief and was confined principally to the election of officers and committees.

Several of the delegates present urged that every possible support be marshaled in aid of the movement now on foot for the establishment of municipal hospitals for the treatment of tuberculosis and for infectious and contagious diseases. Some opposition to the movement has developed, it was said. Institutions now in existence claim that they can care for all such cases at a cost of $10 or $12. A question as to where and how offered by Dr. John H. Pryor was not answered.

"People who can pay $10 or $12 a week don't need a municipal hospital," said Dr. Pryor. "They can go to Colorado or to some other desirable place for treatment. But they are not of the class of people we must reach."

Drs. Pryor, Rochester and Eckel were appointed a committee on hospitals. Mayor Adam, it was stated, would give a hearing within the next two weeks on the proposition to bond the city for $250,000 for the establishment of two hospitals.

Both Dr. Matthew D. Mann, who presided at yesterday's meeting, and Dr. Pryor were nominated for president of the association, but they withdrew in favor of Thomas B. Lockwood, who was unanimously elected. Other officers chosen were: first vice-president, James Carey Evans; second vice-president, Dr. Matthew D. Mann; corresponding secretary, Irving S. Underhill; treasurer, Harry T. Ramsdell.

Charles B. Sears and Chauncey J. Hamlin were elected a committee on incorporation. Frederic Almy, Dr. DeLancey Rochester and Dr. John H. Pryor were named as a committee on constitution and by-laws, and empowered to enlarge the committee by adding to it the names of representatives of various organisations interested in the movement who were not present yesterday, but who took part in the preliminary meeting on December 10, 1908.

The Mayor's approval of the resolution empowering a commission of seven to make a study of details connected with the construction of the two hospitals was sent to City Clerk Balliett yesterday. The resolution came from the committee on sanitary measures and followed a recommendation by the Mayor. The commission is to consist of the Mayor, Commissioner Ward, Health Commissioner Wende, the president of the Common Council, president of the Board of Aldermen, president of the Board of Councilmen and the chairman of the committee on sanitary measures.

Mayor Adam will call a meeting of the commission as soon as the boards are organised. They will visit other cities to inspect municipal institutions, such as are proposed for Buffalo.

